# Emotion Regulation as a Mediator in the Relationship Between Cognitive Biases and Depressive Symptoms in Depressed, At-risk and Healthy Children and Adolescents

**DOI:** 10.1007/s10802-021-00814-z

**Published:** 2021-04-16

**Authors:** A. Sfärlea, K. Takano, C. Buhl, J. Loechner, E. Greimel, E. Salemink, G. Schulte-Körne, B. Platt

**Affiliations:** 1 Department of Child and Adolescent Psychiatry, Psychosomatics and Psychotherapy, University Hospital, LMU Munich, Munich, Germany; 2 Department of Clinical Psychology and Psychotherapy, LMU Munich, Munich, Germany; 3 Deutsches Jugendinstitut (DJI), Munich, Germany; 4 Department of Clinical Psychology, Utrecht University, Utrecht, The Netherlands

**Keywords:** Attention bias, Interpretation bias, Depression, Youth, Eye-tracking

## Abstract

Contemporary cognitive models of depression propose that cognitive biases for negative information at the level of attention (attention biases; AB) and interpretation (interpretation biases; IB) increase depression risk by promoting maladaptive emotion regulation (ER). So far, empirical support testing interactions between these variables is restricted to non-clinical and clinical adult samples. The aim of the current study was to extend these findings to a sample of children and adolescents. This cross-sectional study included 109 children aged 9–14 years who completed behavioural measures of AB (passive-viewing task) and IB (scrambled sentences task) as well as self-report measures of ER and depressive symptoms. In order to maximize the variance in these outcomes we included participants with a clinical diagnosis of depression as well as non-depressed youth with an elevated familial risk of depression and non-depressed youth with a low familial risk of depression. Path model analysis indicated that all variables (AB, IB, adaptive and maladaptive ER) had a direct effect on depressive symptoms. IB and AB also had significant indirect effects on depressive symptoms via maladaptive and adaptive ER. These findings provide initial support for the role of ER as a mediator between cognitive biases and depressive symptoms and provide the foundations for future experimental and longitudinal studies. In contrast to studies in adult samples, both adaptive as well as maladaptive ER mediated the effect of cognitive biases on depressive symptoms. This suggests potentially developmental differences in the role of ER across the lifespan.

## Introduction

Depression is one of the most common psychiatric disorders with a lifetime prevalence of around 20% (Hasin et al., 2018). It affects more than 300 million people worldwide (World Health Organisation, 2018) and by 2030 is expected to be the leading cause of disease (World Health Organisation, 2004). Depression is characterised by persistent low mood combined with disturbances in cognition (e.g., hopelessness about the future) and physical symptoms (e.g., difficulties sleeping; American Psychiatric Association, 2013). Cognitive models underline the role of emotion regulation (ER) difficulties in the development and maintenance of depression. ER has been defined as “the processes by which individuals influence which emotions they have, when they have them, and how they experience and express these emotions” (Gross, 1998, p. 275). ER strategies can be cognitive (e.g., reappraisal of the cause of the emotion) or behavioural (e.g., distraction from the negative emotion via a different activity). A “vicious cycle” between negative emotions, cognitions and behaviours is thought to be responsible for the development and maintenance of depression. Supporting depressed individuals to use more adaptive ER strategies is a core component of many psychological interventions (Young et al., 2019). However, whereas these treatment approaches assume ER is under volitional control, there is a growing awareness of the role that automatic cognitive processes, which are not necessarily under patients’ volitional control, play in ER (Joormann & Stanton, 2016; Wadlinger & Isaacowitz, 2011). The focus of this article is on the extent to which cognitive biases and ER interact in youth depression.

Depression most commonly onsets during adolescence (Hankin et al., 1998) and adolescent depression is associated with greater chronicity (Kovacs, 1996; Lewinsohn et al., 1999; Weissman et al., 1999) and suicide risk (Harrington et al., 1990; Weissman et al., 1999). Childhood and adolescence is a time of ongoing social, cognitive and neurobiological development (Pfeifer & Blakemore, 2012), questioning the appropriateness of directly applying adult aetiological models to youth (Lakdawalla et al., 2007). Whereas negative emotions are regulated by the caregiver in early childhood, adolescence sees a reduction in the reliance on caregivers for ER (Young et al., 2019). Nevertheless, adaptive ER has limited efficacy in modifying negative emotions until early adulthood (Zimmermann & Iwanski, 2014). As such, understanding the association between cognitive biases and ER in youth depression may help to inform improved prevention and treatment methods.

## Emotion Regulation

When faced with events which elicit negative emotions depressed individuals show systematic differences in the ER strategies they habitually use (for reviews see Sheppes et al., 2015; Aldao et al., 2010; Johnstone et al., 2007; D’Avanzato et al., 2013). Depressed individuals are more likely to ruminate (dwell) on the causes of emotional distress (e.g., “what’s wrong with me?”; Nolen-Hoeksema, 2009) and less likely to reappraise the valence, cause or meaning of the event (e.g., “this could give me a chance to pursue other interests and friendships”; Gross, 1998). The fact that the former strategies are positively associated and the latter are negatively associated with depressive symptoms has led to ER strategies being dichotomised as “maladaptive” versus “adaptive”. This dichotomisation is nevertheless controversial (Bonanno & Burton, 2013) since the effectiveness of ER strategies depends partly on the intensity of the stressor (Sheppes & Meiran, 2008; Troy et al., 2013) or its perceived controllability (see Haines et al., 2016). Meta-analyses demonstrate that the effect size (ES) for habitual ER strategies in depressed versus healthy adults is very large: *g’* = 1.12 to 2.10 for maladaptive and *g’* = -0.70 to -1.04 for adaptive strategies (Visted et al., 2018). Experimental and longitudinal studies of adults suggest that ER strategies predict future symptoms of depression and are not simply a by-product of the disorder (Berking et al., 2019; Ochsner et al., 2004).

A meta-analysis of studies of unselected youth found that those with increased depressive symptoms more frequently used maladaptive (and less frequently used adaptive) ER strategies (Schäfer et al., 2017). Increased use of maladaptive ER strategies has also been demonstrated in clinically-depressed youth (Kullik & Petermann, 2013; Sfärlea et al., 2019a). One meta-analysis found that rumination prospectively predicted depressive symptoms in unselected youth (Rood et al., 2009). However, these effects disappeared when baseline symptoms of depression were controlled for (Rood et al., 2009).

## Cognitive Biases

The term cognitive biases refers to quick, automatic cognitive processes which are typically assessed via indirect measures in behavioural paradigms and are biased towards negative (mood-congruent) information (Mathews & MacLeod, 2005). These can occur at the level of attention (attention biases; AB), interpretation (interpretation biases; IB) or memory (Mathews & MacLeod, 2005). For example, when shown an array of emotional stimuli (e.g., faces) depressed individuals and those with elevated symptoms of depression selectively attend to the negative rather than neutral or positive stimuli (Armstrong & Olatunji, 2012; De Raedt & Koster, 2010; LeMoult & Gotlib, 2019). Similarly, depressed individuals also draw more negative interpretations of emotionally ambiguous information. Meta-analyses have shown medium ES for negative AB (Hedge’s *g’* = 0.46 – 0.80; Armstrong & Olatunji, 2012; Peckham et al., 2010) and IB (*g’* = 0.72; Everaert et al., 2017a, 2017b, 2017c) in adult depression. Studies of non-depressed adults (Beevers & Carver, 2003; Sanchez-Lopez et al., 2019), adults with a current (Beevers et al., 2015) or past episode (Browning et al., 2012) of depression suggest negative AB predict future negative mood and depressive symptoms. Similarly, experimental studies support the predictive role of negative IB in explaining negative mood and depressive symptoms (Jones & Sharpe, 2017; Koster & Hoorelbeke, 2015; MacLeod, 2012).

Combined-cognitive bias models of depression (Disner et al., 2011; Everaert et al., 2014) argue that negative AB causally influence negative IB. When attention is easily trapped by negative stimuli, e.g., frowning faces during a talk (AB), this may directly lead to an increase in the negative interpretation of ambiguous scenarios e.g., “they do not like my talk” versus “they cannot hear me well” (IB). This has been supported by experimental studies (Sanchez et al., 2016).

Individual studies show cross-sectional associations between cognitive biases and depressive symptoms in unselected and depressed youth (Kertz et al., 2019; Platt et al., 2017). However, no studies have tested for associations between AB and IB in youth.

## Integrative Models of Depression

Contemporary cognitive models posit a causal effect of cognitive biases on ER (Gross, 1998). For example, according the process model of ER (Gross, 1998), attentional deployment (an antecedent form of ER similar to AB) determines which aspects of an emotional situation will be focused on and thus influences response-focused ER. More specifically, an AB *towards* negative information and/or difficulties *disengaging* from negative information may kindle rumination (repetitive negative thinking; Linville, 1996). Furthermore, negative IB may influence cognitive reappraisal (ER) by consuming the cognitive resources necessary for positively reappraising the event (ERJoormann & D’Avanzato, 2010; Mogg & Bradley, 2018).

The hypothesis that cognitive biases influence ER is supported by a number of empirical studies. Firstly, studies which measure participants’ gaze patterns (AB) when they are given a specific ER strategy demonstrate an *association* (cross-sectional correlation) between AB and the ER strategies reappraisal, suppression and distraction (Bebko et al., 2011; Manera et al., 2014; Strauss et al., 2016; van Reekum et al., 2007). Note that two studies found that the effects of cognitive reappraisal on emotion reactivity were independent of attentional processes (Bebko et al., 2014; Urry, 2010). As Sanchez et al. (2016) note, this may be because reappraisal is influenced by multiple factors (﻿Morris et al., 2014). Secondly, adults who report more frequent rumination also show a negative AB (Duque & Vázquez, 2015; Joormann et al., 2006; Owens et al., 2016) or IB (Everaert et al., 2020; Mor et al., 2014). Thirdly, unselected students who were trained to adopt a positive AB later showed more frequent cognitive reappraisal and more positive mood (Sanchez et al., 2016). Finally, two studies have found that children who ruminate showed a negative AB (Hilt & Pollak, 2013; Romens & Pollak, 2012).

Models of adult depression go one step further by proposing that ER mediates the effect of cognitive biases on depression, i.e. that the effect of cognitive biases in ER contributes to depressive symptoms (Joormann & Stanton, 2016; Wadlinger & Isaacowitz, 2011). This hypothesis is supported by cross-sectional studies of unselected adults (Everaert et al., 2017a, 2020), a longitudinal study of unselected adults (Sanchez-Lopez et al., 2019) and a longitudinal study of adults with a self-reported history of depression (Yaroslavsky et al., 2019). Together they found that rumination does mediate the effect of cognitive biases on depressive symptoms. Furthermore, Everaert et al. (2017a) found evidence to support the model AB IB ER depressive symptoms in unselected adults. The mediating role of ER is yet to be investigated in currently depressed adults.

The hypothesis that ER mediates the effect of cognitive biases on depressive symptoms is also yet to be investigated in youth samples. The relation between cognitive biases, ER and depression may be different in youth compared to adults. Ongoing neurobiological development of brain regions associated with ER (Pfeifer & Blakemore, 2012) may mean that ER plays *less* of a role in mediating the effects of negative AB on depressive symptoms in childhood and adolescence (Kindt & Van Den Hout, 2001). On the other hand, ER may play an even *more* crucial role in determining the effects of automatic cognitive biases on depressive symptoms during this period due to the enhanced emotional sensitivity for negative information (Paus et al., 2008).

## The Current Study

The aim of this cross-sectional study was to extend the finding that cognitive biases have their effect on depressive symptoms via alterations in ER to a well-characterized sample of youth. Although cross-sectional studies are limited in their ability to infer causal relationships, they provide important foundations for randomised controlled trials which are generally more time- and cost-intensive. Clinically-depressed youth, youth with an elevated familial risk of depression and youth with a low familial risk of depression were included in order to maximise variation (recommended by Everaert et al., 2017a). Since the psychometric properties of behavioural measures of cognitive biases are generally poor (Gibb et al., 2016; LeMoult & Gotlib, 2019), measures which had previously shown good reliability were selected (Platt et al., 2021; Sfärlea et al., 2019b). A measure of ER which included sub-scales for adaptive and maladaptive ER was chosen, since it is unclear whether mediating effects are specific to rumination (Everaert et al., 2017a) or also apply to adaptive ER strategies (Sanchez et al., 2016).

Prior to testing a mediation model three hypotheses about direct associations between variables were tested. Firstly, based on studies of adult and youth samples, depressive symptoms were expected to be associated with a negative AB (Armstrong & Olatunji, 2012; De Raedt & Koster, 2010; Kertz et al., 2019; LeMoult & Gotlib, 2019; Peckham et al., 2010; Platt et al, 2017), negative IB (Everaert, Podina, et al., 2017a, 2017b, 2017c; Platt et al., 2017) and increased maladaptive and decreased adaptive ER (Schäfer et al., 2017; Visted et al., 2018) across the sample. Secondly, based on previous findings in adult samples (Everaert et al., 2017a; Sanchez et al., 2016), a positive association between AB and IB was expected across the sample. Thirdly, based on previous findings from adult (Duque & Vázquez, 2015; Joormann et al., 2006; Mor et al., 2014; Owens et al., 2016) and youth (Hilt & Pollak, 2013; Romens & Pollak, 2012) samples, negative AB and IB were expected to be associated with increased maladaptive (and decreased adaptive) ER strategies across the sample.

**Fig. 1:**
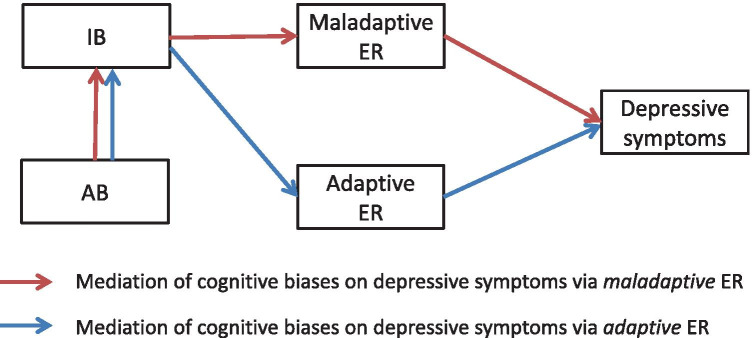
Path model testing mediating role of ER between cognitive biases and depressive symptoms in youth. *Notes*: AB = Attention Bias; IB = Interpretation Bias; ER = Emotion regulation

The fourth hypothesis extended studies of unselected adults (Everaert et al., 2017a) and adults with a history of depression (Yaroslavsky et al., 2019) to youth, and predicted that maladaptive ER strategies would mediate the effects of AB and IB on depressive symptoms. Findings relating to the mediation of AB and IB on depressive symptoms via *adaptive* ER are mixed therefore this pathway was tested but no specific predictions made. Finally, in line with (Everaert et al., 2017a), the mediating role of IB and ER on the relationship between AB and depressive symptoms was tested (Fig. 1).

## Methods

Data were collected within a broader project on cognitive biases in youth depression (Platt, 2017). Data from the same project are reported elsewhere in relation to attention (Platt et al., 2021) and interpretation (Sfärlea et al., 2019b) biases in at-risk youth, as well as interpretation biases in clinically-depressed youth (Sfärlea et al., 2020).

## Participants

Inclusion criteria were participants who were 9–14 years old. Exclusion criteria were intelligence quotient (IQ) < 85 (CFT 20-R; Weiß, 2006), pervasive developmental disorders, attention deficit and hyperactivity disorder, and a history of schizophrenia or bipolar disorder.

In order to maximise variability within the sample (see recommendation by Everaert et al., 2017a), youth had either i) current depression (MD; *n* = 27), ii) no psychiatric disorder but a high familial risk for depression (HR; *n* = 41), or iii) no psychiatric disorder and a low familial risk for depression (LR; *n* = 41).^1^ Diagnoses according to DSM-IV (American Psychiatric Association, 2000) were assessed using standardized, semi-structured psychiatric interviews (K-DIPS; Schneider, Unnewehr, & Margraf, 2009) conducted by trained interviewers with participants and one parent. The K-DIPS has good interrater-reliability (accordance > 96% for all diagnoses; Neuschwander et al., 2013).

The final sample (*n* = 109) excluded youth with poor compliance (2), technical difficulties (3), severe reading difficulties (2), outlying IB scores (2), low quality eye-tracking (ET) data (6), or incomplete questionnaires (2).

Two thirds (72) were female, mean age 12.4 years (*SD* = 1.7) and mean IQ 109.2 (*SD* = 12.1). Three MD participants had recurrent MD, 13 had at least one comorbid anxiety disorder, and two were receiving selective serotonin reuptake inhibitors.

The present data were collected within a study examining IB differences between HR and LR youth using a sample size calculation based on an effect size (ES) *d* = 0.6, α = 0.05 and power = 0.80 (one-tailed test). Accordingly, 36 participants were required per group. For practical reasons we did not quite achieve the required 36 MD participants, but the final sample (*n* = 109) reached the required total sample size and allowed us to detect a significant correlation of r > 0.27 at α = 0.05 and power = 0.80.

The ethics committee of the LMU University Hospital Medical Faculty approved the study. Written informed consent was obtained from participants and parents and participants were rewarded with €30 to €50.^2^ MD participants were recruited though local mental health services. The majority of HR participants were recruited through a study of a preventive intervention for children of depressed parents (Platt et al., 2014) and LR participants were largely recruited through public advertisements.

## Measures

**Attention Bias (AB)**. Eye movements were recorded during a Passive Viewing Task (PVT; Harrison & Gibb, 2015). Stimuli were coloured photographs of children’s faces displaying sad, angry, happy, and neutral emotional expressions from the NIMH Child Emotional Faces Picture Set (NIMH-ChEFS; Egger et al., 2011). The stimulus set comprised 24 models (50% male/female).

**Fig. 2:**
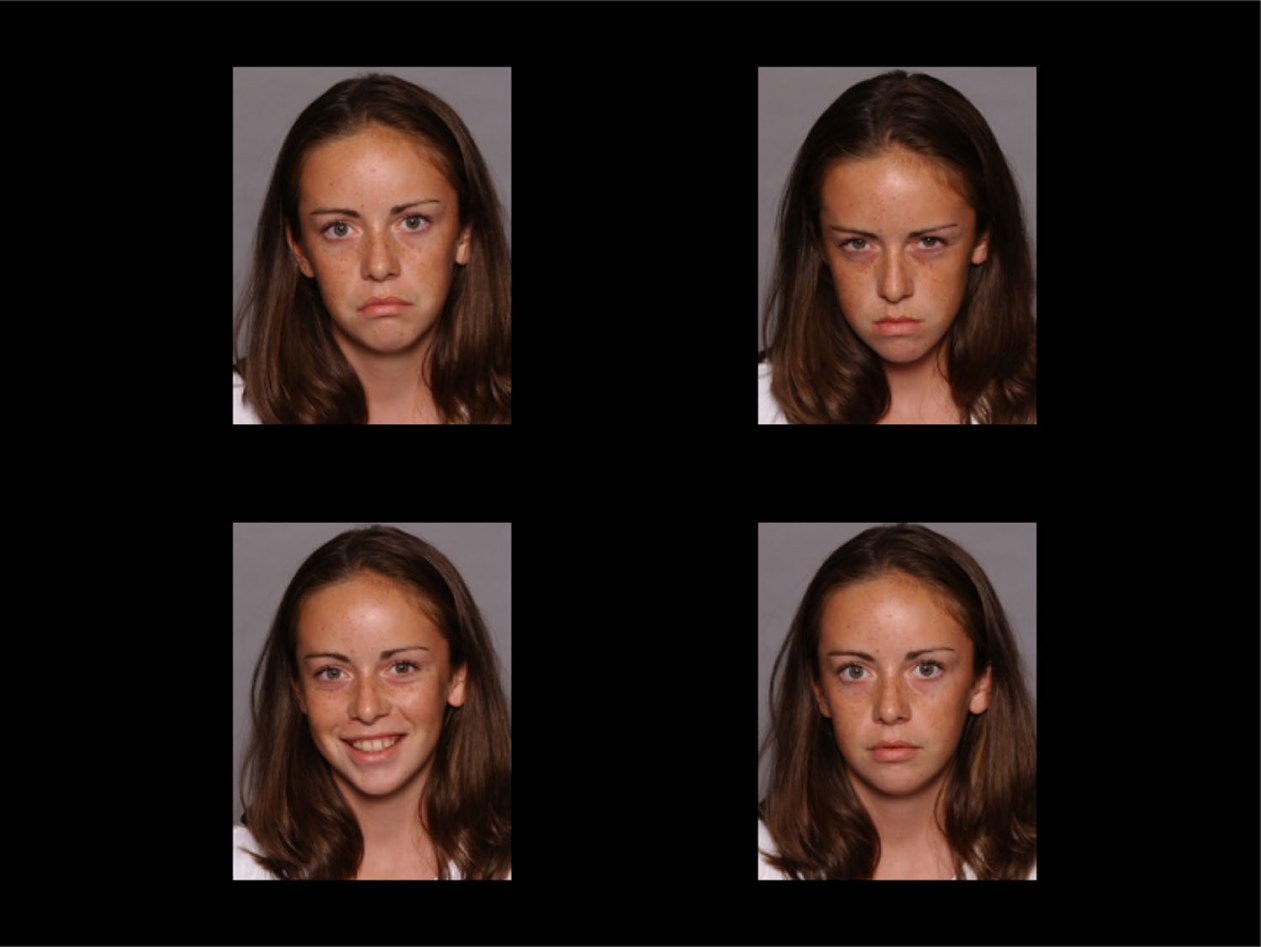
Passive-viewing Task (PVT) stimulus display

Each trial began with a drift correction (small white circle in the centre of the screen). Upon fixation the experimenter initiated the trial. A fixation cross was presented for 1000 ms. Then the 2 × 2 stimulus array was presented for 15,000 ms. The task consisted of 16 emotional trails (corresponding to the minimum trial number suggested for ET research by Orquin & Holmqvist, 2018) and eight neutral trials (not analysed) that were presented in random order. In the emotional trials (Fig. 2), the stimulus array comprised four photographs of the same model displaying a sad, an angry, a happy, and a neutral facial expression. The position of each emotional facial expression was randomly assigned to one of the quadrants with each emotion being presented in each quadrant exactly four times. The neutral filler trails comprised four neutral photographs of the same person. Stimuli had a size of 9.5 × 7.5 cm and were presented with a distance of approximately 6.5 cm horizontally and 1 cm vertically between them. Participants were instructed to freely view the stimuli keeping their attention on the screen.

Eye movements were registered with an EyeLink 1000 Plus desktop-mounted eye-tracker (SR Research, 2013b). Participants were seated in front of a 15inch monitor (1024 × 768 pixel resolution). The experiments were presented using Experiment Builder 1.10 (SR Research, 2013a). Viewing was binocular while eye movements were registered from the dominant eye with a sampling rate of 1000 Hz. A forehead and chin rest were used to minimize head movements and keep the viewing distance (65 cm) constant. The lighting in the room was constant. Before the task commenced, a 9-point calibration and validation procedure was conducted and required average error less than 0.5° of visual angle and maximum error less than 1° of visual angle. Saccades were defined as events with a velocity above 30°/s or an acceleration above 8000°/s^2^ (e.g., Skinner et al., 2018; Waechter et al., 2014). Fixations were defined as gaze positions stable within 1° of visual angle for at least 100 ms (e.g., Duque & Vázquez, 2015). Trials in which the total dwell time was less than 75% of the presentation time (e.g., due to excessive blinks; Skinner et al., 2018) were excluded. The final sample excludes six participants who had poor performance on the AB task (< 70% valid trials; Duque & Vázquez, 2015) or systematic calibration errors. An average of 15.3 trials per participant (*SD* = 1.1; 96% of 16) were available for analysis. AB was defined as mean percentage of dwell time on sad faces due to its good split-half reliability (Spearman-Brown-corrected = 0.81).

**Interpretation Bias (IB)**. A computerized version of the Scrambled Sentences Task (SST; Wenzlaff & Bates, 1998) adapted by Everaert et al. (2014) was used to assess the tendency to form negative or positive statements out of ambiguous information. The stimuli consisted of 30 emotional (e.g., “total I winner a loser am”) and 20 neutral (e.g., “like watching funny I exciting movies”) scrambled sentences (full stimulus set described in Sfärlea et al., 2019b). All sentences contained six words and had two possible solutions. In emotional trials, one solution was positive whereas the other was negative. In neutral trials both solutions were neutral.

**Fig. 3:**
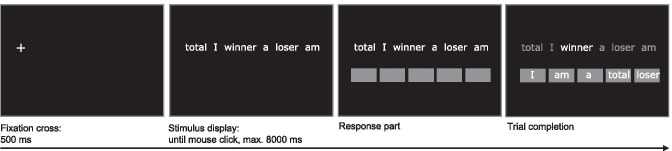
Scrambled Sentences Task (SST) trial procedure

The trial procedure is depicted in Fig. 3. Participants were instructed to read the words, mentally form a grammatically correct five-word sentence as quickly as possible, and click on the mouse button as soon as they did so to continue to the response part of the trial. The scrambled sentence was presented for a maximum of 8000 ms; if no mouse click occurred during that time the response part was omitted and the next trial began. In the response part, five boxes appeared below the scrambled sentence and participants were required to build the sentence they had mentally formed by ordering the words into the five boxes via mouse click. The 50 trials were randomly divided into five blocks of ten, each containing six emotional and four neutral trials presented in random order. Before the first block participants completed five practice trials to familiarize themselves with the task. Similar to earlier studies (e.g., Burnett Heyes et al., 2017; Everaert et al., 2014) a cognitive load procedure was included to prevent deliberate response strategies: before each block, a 4-digit number was presented for 5000 ms which had to be memorized and recalled at the end of the block.

Participants’ responses were rated as correct or incorrect. Trials in which no grammatically correct sentence was built (time-out or incorrect sentence) were excluded. The final sample excludes two participants who had poor performance (< 3 *SD* below the mean). An average of 26.2 correct emotional trials (*SD* = 2.9; 87%) per participant were analysed. IB score was calculated as the proportion of negatively resolved sentences from the total number of correctly resolved emotional sentences (Everaert et al., 2014).

Split-half reliability of the task was assessed by correlating IB scores based on odd versus even trials (see e.g., Van Bockstaele et al., 2017) and was excellent (Spearman-Brown-corrected: 0.94).

The task was administered during ET in order to simultaneously assess AB (Everaert et al., 2014) but the AB index had unacceptable split-half reliability in our sample and was therefore not analysed.

**Emotion Regulation (ER).** The German questionnaire FEEL-KJ (Grob & Smolenski, 2005) contains 90 items assessing self-reported habitual use of seven adaptive and five maladaptive strategies to regulate anxiety, fear, and sadness in youth. Each item is rated on a five-point scale according to how often this strategy is applied to regulate each of the emotions. Sum scores for adaptive and maladaptive strategies across all emotions were calculated. The sum score for adaptive strategies can adopt values from 42 to 210 and the sum score for maladaptive strategies can adopt values from 30 to 150. The questionnaire authors report good internal consistency of the adaptive (Cronbach’s α = 0.98) and maladaptive (Cronbach’s α = 0.82) sub-scales in a sample of unselected children and adolescents (Grob & Smolenski, 2005). Internal consistency in our sample was excellent (Cronbach’s αs ≥ 0.91). Good external validity has been indicated by strong correlations between depressive symptoms and the adaptive (*r* = -0.40, *p* < 0.001) and maladaptive (*r* = 0.35, *p* < 0.001) sub-scales in a large sample of unselected Belgian children and adolescents aged 8 to 18 years (Cracco et al., 2015).

**Depressive Symptoms**. The German version of the Children’s Depression Inventory (DIKJ; Stiensmeier-Pelster et al., 2014) assesses depressive symptom severity and contains 29 items. The DIKJ sum score can vary from 0 to 58 (no clinical cut-offs are available). Good internal consistency of the DIKJ has been reported in a clinical (Cronbach’s α = 0.92) and an unselected (Cronbach’s α = 0.87) sample of youth (Stiensmeier-Pelster et al., 2014). Reliability was excellent in the current sample (Cronbach’s α = 0.96).

## Experimental Procedure

Tasks were administered in random order. As cognitive models of depression suggest that cognitive vulnerabilities such as negative biases are activated by negative mood (e.g. Disner et al., 2011; Scher et al., 2005), a mood induction procedure was administered twice during the experimental session. Participants watched a 2 min scene from the movie *The Lion King* (Hahn et al., 1994) known to successfully induce unpleasant mood in children (von Leupoldt et al., 2007). Participants in this study reported significantly worse mood after watching the movie scene (*t*s ≥ 8.0, *p*s < 0.001). The exact course of the experimental session including the results of the mood induction is reported in Sfärlea et al. (2019b); Supplement 5).

## Data Analysis

Zero-order correlations were calculated to test the first three hypotheses assessing direct relationships across the whole sample between i) each of the cognitive variables (AB, IB, maladaptive ER, adaptive ER) and depressive symptoms, ii) AB and IB and iii) each of the biases and each form of ER. ES are based on Cohen's (1992) recommendation for the interpretation of *r* values: small effect for *r* ≥ 0.10, medium for *r* ≥ 0.30, and large for *r* ≥ 0.50. A path model (see Fig. 1) was generated to test the fourth and fifth hypotheses, which assumed a mediating role of ER in the relationship between iv) cognitive biases and depressive symptoms and v) AB-->IB (mediator) --> ER (mediator) --> depressive symptoms across the whole sample. The path model was estimated using AMOS (Version 25) with the maximum likelihood estimation. Model fit was assessed with Confirmatory Fit Index (CFI) and Root Mean Square Error of Approximation (RMSEA); a model is regarded to have good fit for CFI > 0.95 and RMSE < 0.05. As we did not have a priori hypotheses for the direct effects of AB or IB on depressive symptoms, we performed model selection according to the information criteria, Akaike’s information criterion (AIC) and Bayesian information criterion (BIC), with a lower value indicating better model fit. We started the model selection with the perfect mediation model, where we assumed no direct effect of AB or IB on depressive symptoms. In search of the best-fit model, we added either or both the direct effects (Table 1). If the added direct path(s) improved the model fit, we concluded that the added path has a significant contribution to explain the observed data (and thus should be included in the model). The results of the model selection revealed that the addition of the direct path from IB to depressive symptoms improved the model fit. On this best-fit model with the direct effect of IB, we tested the statistical significance of the individual path coefficients as well as the indirect effects of IB and AB on depressive symptoms via ER. Error intervals of the indirect effects were estimated through bias-corrected bootstrapping with 1000 iterations.

**Table 1:** Model Fit Indices

Model	x^2^	CFI	RMSEA	AIC	BIC
Base model (perfect mediation)	87.87 (*df* = 3, *p* < 0.01)	0.73	0.51	111.87	144.16
With direct effect: IBDepr	2.39 (*df* = 2, *p* = 0.30)	1.00	0.04	28.39	63.38
With direct effect: ABDepr	87.86 (*df* = 2, *p* < 0.01)	0.73	0.63	113.85	148.84
With direct effects: IBDepr ABDepr	2.38 (*df* = 1, *p* = 0.12)	1.00	0.11	30.38	68.06

*AB* attention bias, *AIC* Akaike’s information criterion, *BIC* Bayesian information criterion, *CFI* confirmatory fit index, *Depr.* depressive symptoms, *IB* interpretation bias, *RMSEA* root mean square error of approximation

## Results

Table 2 displays means, SDs and zero-order correlations for the analysed variables.

**Table 2:** Variable correlations (*N* = 109)

Variable	*M*	*SD*	*Min*	*Max*	Correlations			
					1	2	3	4
1. IB	0.25	0.29	0.00	1.00	-			
2. AB	0.23	0.06	0.04	0.59	0.26**	-		
3. Adaptive ER	125.92	34.62	44.00	205.00	-0.50***	-0.25**	-	
4. Maladaptive ER	74.22	20.05	32.00	125.00	0.68***	0.34***	-0.45***	-
5. Depressive symptoms	13.31	12.53	0.00	50.00	0.88***	0.30**	-0.60***	0.77***

*AB* attention bias, *ER* emotion regulation, *IB* interpretation bias, *M* mean; *SD* standard deviation. Correlations remain significant after Bonferroni-Holm (Holm, 1979) correction for multiple testing

*p < 0.05; **p < 0.01; ***p < 0.001

## Hypotheses 1–3

Each of the cognitive vulnerability factors was associated with symptoms of depression (hypothesis 1). Whilst maladaptive ER was positively correlated with AB, IB and depressive symptoms (more negative biases were associated with more maladaptive ER and depressive symptoms), adaptive ER was negatively correlated with AB, IB and depressive symptoms. AB and IB were positively associated (hypothesis 2). AB and IB were positively associated with maladaptive ER and negatively associated with adaptive ER (hypothesis 3).

## Path Analysis—Direct Effects

**Fig. 4:**
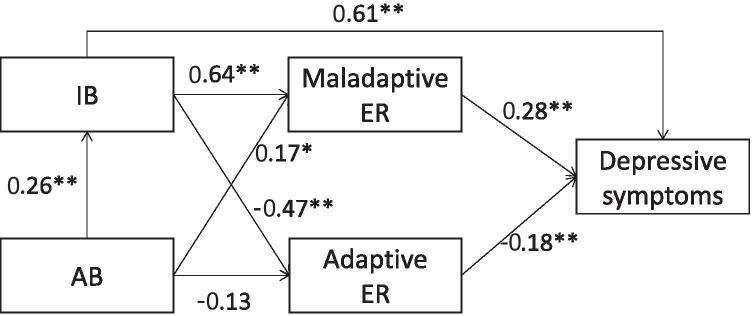
Path model with standardized coefficients. *Notes*. AB = Attention Bias; IB = Interpretation Bias; ER = Emotion regulation. *p < 0.05, **p < 0.01

The estimated path coefficients (direct effects) are shown in Fig. 4. As previously stated, in the model selection procedure it was found that the best model fit did not include the direct effect of AB on depressive symptoms, suggesting that the zero-order correlation observed between AB and depressive symptoms (Table 2) is likely to represent an indirect effect (mediated by another factor). The path model also suggested no direct association between AB and adaptive ER.

## Path Analysis—Mediation (Indirect) Effects

The estimated indirect (mediation) effects testing hypotheses four and five are presented in Table 3, with statistically significant effects indicated when zero was not included in the 95% CI. Maladaptive ER mediated the effect of AB on depressive symptoms and of IB on depressive symptoms (hypothesis 4). Adaptive ER also mediated the effect of IB on depressive symptoms but not of AB on depressive symptoms. The full path effect (AB-->IB-->ER-->depressive symptoms) was significant for both adaptive and maladaptive ER (hypothesis 5).

**Table 3:** Estimated Indirect Effects

Path	Estimates	Lower	Upper
AB-->Maladaptive ER-->Depr.	0.05 *	0.01	0.09
AB-->Adaptive ER-->Depr.	0.02	0.00	0.06
IB-->Maladaptive ER-->Depr.	0.18 *	0.09	0.26
IB-->Adaptive ER-->Depr.	0.08 *	0.04	0.13
AB-->IB-->Maladaptive ER-->Depr.	0.05 *	0.01	0.10
AB-->IB-->Adaptive ER-->Depr.	0.02 *	0.00	0.05

*AB* attention bias, *Depr.* depressive symptoms, *ER* emotion regulation, *IB* interpretation bias

*p < 0.05

## Discussion

### Summary of Results

Cognitive models of depression propose that negative AB and IB increase depression risk by promoting maladaptive ER. The aim of the current study was to extend empirical findings supporting these models in adults to a sample of 109 youth including those with a diagnosis of depression. The data supported a mediating role for both maladaptive *and* adaptive ER. There was also evidence of a direct effect of IB on depressive symptoms.

## Interpretation of Findings

In line with our first hypothesis, all four cognitive constructs were significantly associated with depressive symptoms. The strongest association with depressive symptoms was observed for IB (large ES), supporting a meta-analysis of IB in adult depression (Everaert et al., 2017c). Associations between adaptive and maladaptive ER and depressive symptoms were also of a large ES, supporting meta-analyses of adults (Aldao et al., 2010; Visted et al., 2018) and youth (Schäfer et al, 2017). The medium ES for AB fits with previous literature (Peckham et al., 2010), although our path analysis suggested that the direct effect of AB on depressive symptoms is likely to be minimal. Our second hypothesis was also supported: in line with the combined cognitive hypothesis models of adult depression (Disner et al., 2011; Everaert et al., 2014), we showed for the first time that AB and IB are positively related in youth. Finally, in line with our third hypothesis, we found associations between cognitive biases and adaptive and maladaptive ER. This supports previous (adult) studies of AB and cognitive reappraisal (Bebko et al., 2011; Manera et al., 2014; Strauss et al., 2016; Van Reekum et al., 2007), emotional suppression (Bebko et al., 2011), distraction (Strauss et al., 2016) and rumination (Duque et al., 2014; Joormann, Dkane, Gotlib 2006; Owens et al., 2016). Similarly, it supports studies in adults showing associations between IB and rumination (Everaert et al., 2020; Mor et al., 2014) and cognitive reappraisal (Everaert et al., 2020).

Empirical research testing the hypothesis that ER mediates the effect of cognitive biases on depression has been limited to cross-sectional studies in adults. These studies suggest that rumination (but not cognitive re-appraisal) mediates the effect of negative AB and IB on depressive symptoms (Everaert et al., 2017a; Everaert et al., 2020; Sanchez-Lopez, Koster, Van Put, De Raedt; 2019; Yaroslavsky et al., 2019). Our data also support a mediating role for maladaptive ER. In line with (Everaert et al., 2017a) we also observed a direct effect of IB on depressive symptoms, suggesting that negative interpretations contribute to both depressed mood as well as supporting maladaptive ER. Taken together with findings from studies in adults, our study suggests one subtle difference in the mediating role of ER between adult and youth samples: we found that negative cognitive biases also had an effect on depressive symptoms by hampering *adaptive* ER. As previously stated, in early adolescence children are less reliant on their parents to regulate their emotions, yet adaptive ER is ineffective (Zimmermann & Iwanski, 2014). As a result, they may be particularly sensitive to the effects of negative cognitive biases.

Can ER be considered a mediator in the relationship between cognitive biases and depressive symptoms in youth? The current data give some initial indication to support this idea but further research including longitudinal designs should follow in order to establish causality and the extent to which bi-directional relationships between cognitive biases and ER are in operation. As Kazdin (2007) notes, *temporal precedence* of the cause (cognitive bias) and mediator (ER) over the outcome (depressive symptoms) is needed to truly establish mediation. No longitudinal study has investigated whether changes in cognitive biases predict subsequent changes in ER and depressive symptoms. A second requirement is *experimental manipulation* of the cause and proposed mediator (Kazdin, 2007). Adult studies show that modifying ER is associated with improvements in depressive symptoms (e.g., Berking et al., 2019) and modifying AB results in increased use of cognitive reappraisal (Sanchez et al., 2016). Similar studies in youth samples are needed. Experimental studies may also be helpful in testing bi-directional relationships between the variables. For example, some authors have argued that a tendency to draw negative interpretations directs future attention (Sanchez et al., 2015) and that maladaptive forms of ER may lead to more negative IB (Hilt & Pollak, 2013).

Since *consistency* of findings in multiple studies and samples is another requirement (Kazdin, 2007), our study provides a valuable contribution to the literature by extending findings in adults to a sample of youth including those with clinical depression. The current findings also fulfil the requirement for *strong relationships* (Kazdin, 2007) between IB and ER and between ER and depressive symptoms.

One additional finding which deserves attention is that IB partially mediated the effect of AB on depressive symptoms. This finding replicates a similar cross-sectional study in adults (Everaert et al., 2017a). More broadly the finding also supports combined-cognitive bias models of depression which suggest that when attention is trapped by negative stimuli they cause negative interpretation of ambiguous scenarios (Disner et al., 2011; Everaert et al., 2014). As far as we are aware, this is the first study to demonstrate a significant association between AB and IB in youth and the first study to suggest that IB partially mediates the effects of AB on depressive symptoms in youth.

## Strengths and Limitations

A strength of the current cross-sectional study is that it provides the foundations for future experimental studies designed to determine causal relationships between cognitive biases, ER and depressive symptoms in youth with depression. A major strength of the study was the use of measures with good to excellent psychometric properties, particularly given the generally poor reliability of behavioural and ET measures in the field (Gibb et al, 2016; LeMoult & Gotlib, 2019). The sample also included participants with a clinical diagnosis of depression as well as those with an elevated familial risk of depression. This increases the power of the study to detect effects due to increased variability in the data and improves the external validity of the study. A final strength is the use of standardised psychiatric interviews which had good inter-rater reliability.

As previously mentioned, the cross-sectional nature of the study is a limitation since it prevents causal inferences from being drawn. In addition to experimental studies, longitudinal studies will be helpful in determining the directionality of effects, for example, whether IB influences ER or vice versa. Of note, one cannot rule out the possible influence that top-down processes, such as ER, had on our measures of AB and IB. Although both included a time limit and the SST included a cognitive load procedure, ER processes may nevertheless have influenced participants scores on AB and IB measures. Indeed, we are also unable to rule out the influence that AB and IB had on the scores participants obtained on the self-report ER measures.

The relatively modest sample size is a further limitation of the current study. We also cannot be sure that our findings are specific to symptoms of depression versus anxiety. Thirteen of the 27 MD patients had comorbid anxiety, which is comparable with expected rates of comorbidity (Essau, 2005). When recalculating the path analysis excluding the participants with comorbid anxiety disorders in an exploratory post-hoc analysis, all direct effects between the study variables remained significant except the association between AB and IB.^3^ However, we refrain from interpreting this post-hoc finding since the statistical power of the analysis is compromised (> 10% reduction in sample size) and the variability in depressive symptoms was reduced (just 14 patients were left in the sample). Future studies with larger sample sizes might investigate if this is an artefact or if the AB-IB relationship is indeed dependent on anxiety. However, studies seeking to disentangle the effects of depression and anxiety by directly comparing samples of youth with pure depression versus anxiety are limited since comorbid anxiety is considered the rule rather than the exception in youth depression (Essau, 2005; Nottelmann & Jensen, 1999).

## Clinical Implications

Since most psychotherapeutic interventions for youth depression include elements of ER (Young et al., 2019) the current findings highlight the need for psychotherapists to be aware of the powerful influence that cognitive biases have on ER. Combined with data from studies in adult samples (Everaert et al., 2020; Yaroslavsky et al., 2019) they suggest that if underlying cognitive biases are not addressed in psychotherapy, effective ER may be hampered. Studies which address the extent to which traditional therapeutic approaches modify cognitive biases could be helpful in this endeavour. Furthermore, therapeutic approaches which combine both explicit therapeutic techniques (e.g., cognitive restructuring) as well as automatic cognitive biases (e.g., cognitive bias modification) may be particularly effective. Finally, if negative cognitive biases have their influence on depressive symptoms by hampering adaptive ER (Joormann & D’Avanzato, 2010), then training cognitive biases during situations that evoke negative emotions (and therefore require adaptive ER) may be more effective than training cognitive biases in non-emotional situations.

## Future Research

As previously mentioned, future experimental and longitudinal studies will be helpful. Across study designs we encourage researchers to select measures based on psychometric properties. One important construct in cognitive models of depression which may be useful to include in future studies is cognitive control. People with (high risk of) depression have deficits in cognitive control (Grahek et al., 2018) which may contribute to increased use of rumination, hamper cognitive reappraisal (Cohen & Ochsner, 2018; Joormann & Siemer, 2011; LeMoult & Gotlib, 2019) and heighten negative cognitive biases (Everaert et al., 2017b) by failing to remove irrelevant negative information from working memory (Joormann et al., 2007). Since the relation between cognitive biases, ER and depression may vary across the lifespan, future developmental studies which specifically address age-related changes could be particularly informative.

## Conclusions

Contemporary cognitive models of depression posit that negative cognitive biases influence depression by altering ER processes. We provide the first empirical evidence to support this model in youth. In contrast to the adult literature, in which negative cognitive biases increase the use of *maladaptive* ER, we found evidence that for youth, negative cognitive biases also hamper *adaptive* ER. Our findings require replicating in experimental and longitudinal designs but provide preliminary evidence that interventions designed to improve ER for youth with depression are likely to be enhanced if they also address underlying negative cognitive biases.

## Data Availability

Data will be made publicly available via the Open Science Framework.

## Footnotes

1 Children at high familial risk for depression had at least one parent that had suffered from depression or dysthymia during the child’s lifetime. Children were not included if the affected parent had a history of bipolar disorder, schizophrenia, or substance abuse but other current or past comorbidities were allowed. Of some children both parents were affected by depression but psychiatric diagnosis was not systematically assessed in the second parent. Children at low familial risk for depression had parents with no history of any mental disorder. Both parents’ psychopathology was considered for these participants, whenever possible. Parental psychopathology was assessed with a standardized psychiatric interview (DIPS; Schneider & Margraf, 2011).

2 HR and LR participants who participated in the study with their parents received a reimbursement of €50 per family while participants taking part without their parents received a reimbursement of €30.

3 With the 13 patients with anxiety disorder removed the direct effect of AB IB was 0.12 (n.s.). Accordingly, the indirect paths i) AB-->IB-->Maladaptive ER-->Depr. and ii) AB-->IB-->Adaptive ER-->Depr. were no longer significant. The overall model fit was reduced (χ^2^ = 7.43, *p* = 0.042; CFI = 0.975; RMSEA = 0.169).

## Code Availability

Code will be made publicly available via the Open Science Framework.
